# Complications de la stimulation cardiaque définitive: étude observationnelle, rétrospective à propos de 462 cas au Centre Hospitalier et Universitaire Hedi Chaker de Sfax, Tunisie

**DOI:** 10.11604/pamj.2024.49.24.25891

**Published:** 2024-09-30

**Authors:** Rahma Kallel, Rania Hammami, Aiman Dammak, Faiza Safi, Malek Akrout, Leila Abid, Samir Kammoun, Jedidi Jihen

**Affiliations:** 1 Service de Cardiologie, Centre Hospitalier Universitaire Hedi Chaker de Sfax, Sfax, Tunisie,; 2 Service de Chirurgie Cardiovasculaire, Centre Hospitalier Universitaire Habib Bourguiba, Sfax, Tunisie,; 3 Service de Réanimation Pédiatrique, Centre Hospitalier Universitaire Hedi Chaker de Sfax, Comité Pédagogique, Faculté de Médecine de Sfax, Sfax, Tunisie,; 4 Service d'Epidémiologie et Médecine Communautaire, Centre Hospitalier Universitaire Hedi Chaker de Sfax, Sfax, Tunisie

**Keywords:** Pacemaker, défibrillateur, complications, endocardite infectieuse, hématome, déplacement de sonde, dysfonction de sonde, pneumothorax, tamponnade, mortalité, Pacemaker, defibrillator, complications, infective endocarditis, haematoma, lead displacement, lead dysfunction, pneumothorax, tamponade, mortality

## Abstract

Le bénéfice de la stimulation cardiaque définitive a été largement démontré. Cependant, les données de littérature concernant les complications sont divergentes. Nous n'avons pas d'idée précise concernant les fréquences des complications ni leurs facteurs prédictifs dans notre centre. Nous nous proposons de déterminer les fréquences des complications liées à la stimulation cardiaque définitive dans notre structure et de préciser leurs facteurs prédictifs. Il s'agit d'une étude rétrospective, observationnelle descriptive et analytique. Elle concernait les patients ayant bénéficié d'une procédure sur dispositif électronique implantable en cardiologie (DEIC) soit, pacemaker (PM) ou défibrillateur implantable (DAI) entre janvier 2009 et décembre 2013 au centre hospitalo-universitaire de Sfax, Tunisie. Toutes les caractéristiques cliniques et paracliniques des patients, leurs données procédurales ainsi que les éventuelles complications liées à l'implantation des DEIC ont été recueillies (les complications infectieuses, les hématomes de la loge, les complications liées aux sondes, celles liées à l'abord vasculaire et la mortalité en cas de complication). Les tests statistiques appropriés ont permis d'analyser les incidences des complications, leurs facteurs associés grâce à l'analyse multivariée et d'étudier la survie. Nous avons collecté les données de 462 procédures, dont 420 PM et 42 DAI. La population était d'âge moyen 72 ans ± 15. Ils étaient hypertendus dans 55,1% des cas, diabétiques dans 22,3% des cas et 63,38% avaient une cardiopathie sous-jacente. Soixante-quatre complications étaient notées soit 11,5% des procédures. Les complications étaient significativement plus fréquentes avec les DAI que les PM (23,8% Vs 10,2%; p=0,04). L'incidence des complications infectieuses était de 1,96%. Ses facteurs de risque associés étaient le diabète (ORa: 4,35, 95% IC 1,08 -17,48; p=0,038) et fraction d'éjection ventriculaire altérée (ORa:9,2, 95% IC 1,83-46,12; p=0,007). L'incidence des hématomes de la loge était de 1,53%, son facteur de risque associé était l'indication à une anticoagulation curative (ORa:29,05, 95% IC 3,42-246,57; p=0,002). Les complications liées aux sondes étaient les plus fréquentes (73,4% des complications). Leur facteur prédictif indépendant était le nombre de manipulations >à 1 (ORa: 3,66, 95% IC 0,98-13,61; p=0,05). Parmi ce sous-groupe, le déplacement de sonde était le plus fréquent (40,05%) ayant comme facteur de risque associé, la présence d'une cardiopathie hypertensive (ORa: 3,99, 95% IC 1,2-13,1; p=0,019). Les complications liées à l'accès vasculaire étaient rares, 0,21% des cas. La mortalité liée aux complications des stimulateurs cardiaques était élevée (13,2%), en particulier en cas de complication infectieuse (p=0,04). La survie globale à 5 ans était à 84,5%. L'incidence des complications des DEIC à court et à long terme dans notre centre était haute avec une mortalité conséquente considérable mais ça reste comparable aux données de la littérature. Grâce à l'identification des facteurs de risque associés comme le diabète, l'insuffisance cardiaque, l'anticoagulation curative et la reprise opératoire, nous pouvons adopter une attitude thérapeutique avisée pour réduire les complications.

## Abstract (en)

The benefits of permanent cardiac pacing have been widely demonstrated. However, the literature on complications remains inconsistent. We lack precise information about the frequency of complications and their predictive factors in our center. The purpose of this study was to determine the frequency of complications related to permanent cardiac pacing in our centre and to specify their predictive factors. We conducted a retrospective, observational, descriptive and analytical study. It involved patients who underwent an implantable electronic device (CIED) procedure, such as a pacemaker (PM) or implantable cardioverter-defibrillator (ICD) at the University Hospital Center of Sfax, Tunisia between January 2009 and December 2013. All clinical and paraclinical characteristics of the patients, their procedural data and any potential complications related to CIED implantation were collected (infectious complications, pocket hematomas, lead-related complications, vascular access complications, and complication-related mortality). Appropriate statistical tests were used to analyze the incidence of complications and their associated factors through multivariate analysis and to perform a survival analysis. We collected data from 462 procedures, including 420 PMs and 42 ICDs. The population had an average age of 72 ± 15 years. Hypertension was present in 55.1% of cases, diabetes in 22.3%, and 63.38% had underlying heart disease. A total of 64 complications were noted, accounting for 11.5% of the procedures. Complications were significantly more frequent with ICDs than PMs (23.8% vs. 10.2%; p=0.04). The incidence of infectious complications was 1.96%. Associated risk factors included diabetes (adjusted OR: 4.35, 95% CI 1.08-17.48; p=0.038) and reduced left ventricular ejection fraction (adjusted OR: 9.2, 95% CI 1.83-46.12; p=0.007). The incidence of pocket hematomas was 1.53%, with its associated risk factor being an indication for therapeutic anticoagulation (adjusted OR: 29.05, 95% CI 3.42-246.57; p=0.002). Lead-related complications were the most common (73.4% of complications). Their independent predictive factor was the number of manipulations greater than one (adjusted OR: 3.66, 95% CI 0.98-13.61; p=0.05). Among this subgroup, lead displacement was the most frequent (40.05%), with the presence of hypertensive heart disease as an associated risk factor (adjusted OR: 3.99, 95% CI 1.2-13.1; p=0.019). Vascular access complications were rare, occurring in 0.21% of cases. Mortality related to complications of cardiac device implantation was high (13.2%), particularly in the case of infectious complications (p=0.04). Overall survival at 5 years was 84.5%. The incidence of IEDC-related complications in the short and long term at our center was high, with a significant associated mortality, although comparable to the literature data. By identifying associated risk factors such as diabetes, heart failure, therapeutic anticoagulation, and repeat surgeries, we can adopt an informed therapeutic approach to reduce complications.

## Introduction

L'implantation des stimulateurs cardiaques est une thérapie en plein essor avec une évolution récente des techniques de stimulation imposant progressivement une évolution des pratiques [1]. L'élargissement des indications à la stimulation définitive par pacemaker (PM) ou défibrillateur automatique implantable (DAI) est à l'origine de l'expansion des procédures d'implantations et donc de leurs complications [2,3]. Par ailleurs, le taux de complications est inversement proportionnel aux volumes d'implantations individuels et de centres [3,4]. Le spectre de ces complications est large [4], variant avec les prédispositions des malades et les particularités liées aux procédures et les différents types de dispositifs électroniques implantables en cardiologie (DEIC). Certaines complications sont graves s'accompagnant d'une surmortalité importante [5]. Ainsi, la prolongation du séjour hospitalier et les coûts supplémentaires sont un fardeau considérable à la santé [4,5]. Les données de la littérature concernant ce sujet sont très variables voire divergentes [2,3,5]. Par ailleurs, en l'absence de registre dans nos structures hospitalières, nous ne disposons pas de recul concernant notre expérience. D'où la nécessité de préciser l'ampleur de ces complications, et surtout les facteurs associés, qui peuvent être modifiables ou non [5], afin d'adopter des mesures préventives avisées à ces complications. Nous nous proposons à travers ce travail de déterminer la fréquence des complications survenant à court et à long terme après implantation de PM ou DAI dans notre centre. Notre deuxième objectif est de déterminer les facteurs prédisposants à la survenue de chaque complication.

## Méthodes

**Concept de l'étude:** il s'agit d'une étude observationnelle rétrospective descriptive et analytique. Elle a été réalisée au centre hospitalo-universitaire (CHU Hedi Chaker) situé à Sfax, Tunisie.

**Population d'étude:** elle a porté sur la totalité des procédures d'implantation de stimulateur cardiaque type pacemaker ou défibrillateur, dans notre étude pendant une période de 5 ans s'étalant entre janvier 2009 et décembre 2013. Les critères de non inclusion étaient: Les complications des stimulations temporaires, les complications des implantations épicardiques et les complications des patients ayant bénéficié de la dernière procédure dans un autre centre.

**Collecte des données:** elle a été faite à partir des dossiers médicaux des patients, des registres de la salle de cathétérisme cardiaque et des comptes rendus échographiques. Nous avons systématiquement renseigné toutes les données démographiques, cliniques et paracliniques liées à la procédure index et éventuellement à la complication en question. Nous avons contacté les patients ou leurs médecins par téléphone pour récupérer les informations manquantes chaque fois que c'était possible.

**Définitions:** nous avons adopté le regroupement des complications des dispositifs électroniques implantables conformément aux plus récentes recommandations européennes de cardiologie [2,3]. Nous avons retenu le délai d’un an pour définir les complications infectieuses précoces [6] et 12 semaines pour les complications non infectieuses précoces. Après ces délais les complications sont considérées tardives [3]. Concernant les complications infectieuses, nous avons distingué ces deux entités conformément aux recommandations récentes [2,5]. L'endocardite sur stimulateur cardiaque: une infection «systémique» étendue aux sondes, aux valves et à la surface de l'endocarde. L'infection de la loge: elle se limite à des signes inflammatoires locaux à type de rougeur, chaleur, flatulence, désunion des sutures, issue de sérosités voire du pus et même l'érosion et l'extériorisation du boitier.

**Analyse statistique:** la saisie et l'analyse des données étaient faites par le logiciel SPSS Inc version 20. Concernant la partie descriptive, nous avons utilisé des effectifs et des pourcentages pour la description des variables qualitatives. Pour les variables quantitatives, nous avons calculé des moyennes et des écarts-types lorsque la distribution de la variable était gaussienne, des médianes et des extrêmes dans le cas contraire. En ce qui concerne l'étude analytique, nous avons utilisé le test de chi^2^ de Pearson sur séries indépendantes pour la comparaison de deux fréquences lorsque les conditions d'application étaient vérifiées, et le test exact bilatéral de Fisher dans le cas contraire. La comparaison de 2 moyennes sur séries indépendantes a été effectuée par le test T de Student sur séries indépendantes lorsque les conditions d'application étaient vérifiées, et par le test de Mann-Whitney dans le cas contraire. L'analyse de la corrélation entre la croissance de la fréquence des complications par an avec la croissance des taux d'implantation a été faite par le test de corrélation de Spearman. Le seuil de significativité était fixé à 5%.

Pour l'analyse multivariée, nous avons utilisé le test de régression logistique binaire. Nous avons introduit dans le modèle toute variable ayant une signification inférieure ou égale à 0,2. Pour l'analyse de la mortalité liée aux complications, seules les données des patients ayant présenté une complication étaient étudiées. L'étude étant faite par l'analyse des courbes de survie de Kaplan Meyer. La date de point de l'étude était fixée à juin 2014. La comparaison des courbes de survie était faite par le test de Logrank, et le seuil de significativité était fixé à 5%.

**Les considérations éthiques:** nous avons respecté les données personnelles des patients. Les patients contactés pour complément d'information dans le cadre de recherche scientifique ont accepté de répondre et étaient très coopérants.

## Résultats

**Caractéristiques générales de la population d'étude (Tableau 1):** notre étude a inclus 462 procédures sur dispositif électronique implantable (DEIC) dont 420 PM et 42 DAI. Ces dispositifs étaient de type mono-chambre dans 51,2% des cas, double chambre dans 43,2% et triple chambre dans 5,6% des cas. La population avait un âge moyen de 72±15 ans, était de sexe féminin dans 50% des cas, hypertendus dans 55,1% des cas, et diabétiques dans 22,3% des cas. Une cardiopathie sous-jacente a été identifiée dans 63,38% des cas. Les cavités droites étaient dilatées dans 18% des cas. Nous avons observé 64 complications soit 11,5% des procédures (Tableau 2). Le taux de complications était significativement plus important avec les DAI (23,8%) qu'avec les PM (10,5%), p=0,01.

**Tableau 1:** caractéristiques de la population étudiée

Age moyen (écart type)	72 ans±15
Sexe masculin	50%
HTA	55,1%
Diabète	22,3%
Tabac	19%
IRC	4,3%
Cardiopathie sous-jacente	63,38%
Fraction d'éjection du VG abaissée <50%	27,2%
Cavités droites dilatées	18%
HTAP >40 mmHg	27,7%
DEIC monochambre	51,2%
DEIC double chambre	43,2%
DEIC triple chambre	5,6%

HTA: hypertension artérielle, IRC: insuffisance rénale chronique, VG: ventricule gauche, HTAP: hypertension artérielle pulmonaire, DEIC: dispositif électronique implantable en cardiologie

**Tableau 2:** récapitulatif des complications des dispositif électroniques implantables en cardiologie

Complications liées à	Entités	DEIC (N=462) n (%)	PM			DAI		
			Total PM (N=420) n(%)	Mono et double (N=404) n(%)	Triple chambre (N=16) n(%)	Total DAI (N=42) n(%)	Mono et double (N=32) n(%)	Triple chambre (N=10) n(%)
La voie d'accès	Pneumothorax	1 (0,21%)	1 (0,21%)	1 (0,2%)	0	0	0	0
Infections	EI/Infection loge	9 (1,96%)	6 (1,5%)	6(1,5%)	0	3(7,1%)	0	3 (30%)
La loge	Hématomes	7 (1,5%)	6	6 (1,5%)	0	1(2,3%)	0	1(10)
Les sondes	Arythmies	8 (1,7%)	3 (0,7%)	3 (0,7%)	0	5 (11,9)	4 (12,5)	1 (10%)
	Perforation cardiaque	1 (0,21%)	1	1 (0,21%)	0	0	0	0
	Dissection du sinus coronaire	2 (0,4%)	1 (0,2%)	0	1 (6,2%)	1(4,7%)	0	1 (10%)
	Déplacement	19 (4,1%)	17 (4%)	17(4,2%)	0	1(2,4%)	0	1
	Dysfonction	3 (0,6%)	3 (0,7%)	3 (0,7%)	0	1(2,4%)	1 (3,1)	0
	Thromboembolisation	8 (1,7%)	8 (1,9%)	8 (2%)	0	0	0	0
	Echec d'implantation	4 (0,9%)	3 (0,7%)	3 (0,7%)	0	1(2,4%)	0	1 (10%)
	Syndrome du PM	2 (0,4%)	2 (0,5%)	2 (0,5%)	0	0	0	0
	Total	47	38	37	1	9	5	4
Total complications N (%)		64(13,85)	51(12,14)	50(12,37)	1(6,25)	13(30,95)	5(15,62)	8(80)

DEIC: dispositif électronique implantable en cardiologie; PM: pacemaker; DAI: défibrillateur automatique implantable; EI: endocardite infectieuse

**Les complications infectieuses:** nous avons enregistré 9 cas (1,96%), de type endocardite infectieuse dans 7 cas et infection de loge dans 2 cas. Ces infections étaient précoces dans 5 cas. D'après les résultats de l'analyse univariée, les facteurs liés au malade étaient l'âge inférieur à 70 ans, le diabète, la dysfonction ventriculaire gauche (FEVG<50%) et la cardiopathie ischémique sous-jacente (Tableau 3). Les facteurs non liés au malade étaient le type DAI et le nombre de sondes supérieur ou égal à deux (Tableau 4). L'affinement de nos résultats par l'analyse multivariée a permis de retenir le diabète (ORa:4,35, 95% IC 1,08 -17,48; p=0,038) et la FE VG <50% (ORa:9,2, 95% IC 1,83-46,12; p=0,007) comme des facteurs associés aux complications infectieuses.

**Tableau 3:** étude des facteurs prédictifs des complications infectieuses liés au patient

		Population	Survenue de complications infectieuses		OR (95%IC)	p	ORa (95%IC)	p
			N	%				
Age <70 ans		Oui (N=152)	7	4,6	5,8(1,17-33,3)	0,03	-	-
		Non (N=238)	2	0,8				
Sexe		Masculin (N= 230)	4	1,7	0,73	-	-	-
		Féminin (N=232)	5	2,2				
Diabète		Oui (N= 84)	5	6	4,7(1,2-17,2)	0,01	4,35 (1,08-17,48)	0,038
		Non (N= 292)	4	1,4				
HTA		Oui (N= 207)	4	1,9		0,75	-	-
		Non (N= 169)	5	3				
Tabac		oui (N= 88)	4	4,5		0,22	-	-
		Non (N= 288)	5	1,7				
Présence de cardiopathie sous-jacente		Oui (N= 180)	8	4,4		0,16	-	-
		Non (N= 104)	1	1				
FE VG		Normale (N=206)	2	1		0,002	9,2 (1,83 -46,12)	0,007
		Abaissée (N=77)	7	9,1	10(2,07-50,2)			
Cardiopathie	Valvulaire	Oui (N= 29)	1	3,4	1		-	-
		Non (N= 255)	8	3,1				
	Hypertensive	Oui (N=58)	3	5,2	0,39		-	-
		Non (N= 226)	6	2,7				
	Ischémique	Oui (N=44)	4	9,1	4,6(0,8-22,6)	0,035	-	-
		Non (N= 240)	5	2,1				
HTAP		Oui (N= 78)	5	6,4		0,12	-	-
		Non (N= 204)	4	2				

HTA: hypertension artérielle, FEVG: fraction d'éjection du ventricule gauche, HTAP: hypertension artérielle pulmonaire, DEIC: dispositif électronique implantable en cardiologie, OR: odds ratio, ORa: odds ratio ajusté, IC: intervalle de confiance

**Tableau 4:** étude des facteurs non liés au malade et prédictifs de complications infectieuses

	Population	Complications de DEIC		OR (95%IC)	p	OR ajusté (95%IC)	p
		N	%				
**Type de dispositif**	DAI (N= 42)	3	7,1	5,3(1,27-22,5)	0,04	-	-
	PM (N= 420)	6	1,4				
**Nombre de sondes**	Triple chambre (N= 26)	3	11,5	9,34 (2,19-39,77)	0,01	-	-
	Mono ou double chambre (N=436)	6	4,9				
**Voie d'abord**	Sous clavière (N= 327)	9	14,28	-	0,36	--	--
	Céphalique (N=63)	00	00				
**Antibioprophylaxie**	Céphalosporines (N=402)	9	2,23	-	1	-	-
	Glycopeptides (N=38)	00	00				
**Anticoagulation**	Oui (N= 57)	1	1,8	-	1	-	-
	Non (N=248 )	8	3,2				

DEIC: dispositif élèctronique implantable en cardiologie, PM: pacemaker, DAI: défibrillateur automatique implantable, OR: odds ratio, ORa: Odds Ratio ajusté, IC: intervalle de confiance

**Les complications non infectieuses:** l'hématome de la loge nécessitant la réintervention était précoce, noté dans 7 cas (1,53%). Selon l'analyse univariée, les facteurs associés étaient le tabagisme, les antécédents thromboemboliques, le chevauchement héparine-AVK et le traitement par AVK. Après l'analyse multivariée, seulement le traitement par AVK était significativement associé (ORa:29,05, 95% IC 3,42-246,57; p=0,002). Les complications liées aux sondes étaient les plus fréquentes (73,34%). Les facteurs prédictifs de ces complications étaient en univariée: le sexe masculin, la présence de cardiopathie hypertensive, le DEIC à type de DAI et le nombre de manipulations >1. Après analyse multivariée, seulement le nombre de manipulations >1 était significativement associé aux complications liées aux sondes (ORa:3,66, 95% IC 0,98 -13,61; p=0,05). Parmi ce groupe, le déplacement des sondes a constitué la complication la plus fréquente (40,05%). Les facteurs prédisposant au déplacement de sondes étaient en univarié, le tabagisme et la cardiopathie hypertensive et en analyse multivariée: la cardiopathie hypertensive (ORa:3,99, 95% IC 1,2-13,1; p=0,019). Les autres complications liées aux sondes étaient rares à savoir les dysfonctions de sondes (0,64% des cas), les échecs d'implantation (1,05% des cas). Un cas de perforation (0.21%), la thrombose de la veine sous clavière (0.21%). Les complications liées à l'abord vasculaire se résumaient en un cas de pneumothorax (0,21%).

**Figure 1:**
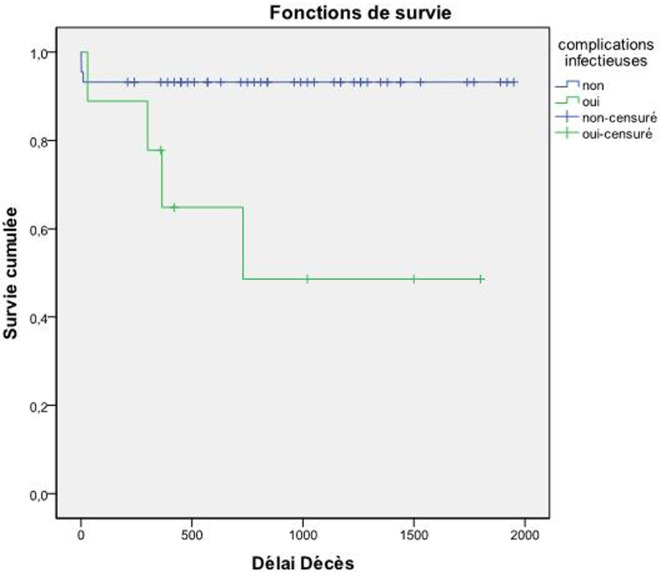
courbe de survie des patients avec complication infectieuse

**La mortalité liée aux complications de DEIC:** elle a atteint 13,2%, la survie globale à 5 ans était de 85,4%. Parmi ces complications, les complications infectieuses étaient associées de façon significative à une surmortalité à 5 ans (p=0,004) (Figure 1).

## Discussion

Au moment où une nouvelle ère de stimulation cardiaque s'annonce avec la stimulation septale et du système de conduction [1,2], l'accès à ces techniques reste encore limité sous nos cieux et une meilleure maitrise des complications liée à la stimulation classique reste toujours d'actualité. Pour ce, il est crucial d'évaluer notre pratique, en déterminant, les incidences des complications majeures et leurs facteurs associés. Nous avons focalisé l'étude rétrospective sur une période de 5 ans (2009-2013). Les données de 462 procédures sur pacemakers (n=420) et défibrillateurs (n=42) avec leurs suivis ont été collectées. La population était âgée (72 ± 15 ans) avec hypertension artérielle dans 55,1% des cas, cardiopathie sous-jacente dans 63,38% des cas. Le taux global des complications était 11,5%. Les complications infectieuses ont touché 1,96% des procédures, significativement plus fréquentes chez les diabétiques (ORa=4,35) et en cas de fraction d'éjection ventriculaire abaissée (ORa=9,2). Ce type de complication a été associé à une surmortalité significative. Les hématomes de loge réopérés ont compliqué 1,53% des procédures avec un facteur associé l'anticoagulation aux AVK (ORa=29,05). Les complications liées aux sondes étaient les plus fréquentes (73,4%), en particulier le déplacement de sonde. Ces complications sont plus fréquentes en cas de nombre de manipulations >1. La mortalité globale était élevée en cas de complication (13,4%). Le taux global de complications était comparable aux données de la littérature avec des taux entre 11 à 16% [7]. FOLLOWPACE a rapporté une fréquence de complications de 12,4%. Les complications infectieuses constituent la hantise majeure de la stimulation définitive car elle est associée à une surmortalité [5,8], retrouvée dans notre travail. L'incidence des complications infectieuses est très variable selon les études [5]. La revue de 22 études comportant chacune plus de 1000 patients, a montré qu'elle range entre 0,5% et 2,2% [8]. Dans notre centre, la fréquence des complications infectieuses était (1,96%) comparable aux données de la littérature. De même pour les facteurs associés aux complications infectieuses, à savoir, le diabète et la dysfonction ventriculaire (FE VG <50%). En effet, les facteurs prédictifs selon la littérature étaient essentiellement: le diabète, la présence de cardiopathie sous-jacente [9,10], les DAI en comparaison avec les PM [11], le nombre de sondes implantées de plus de deux [5,9,12] et la réintervention précoce avec un OR à 15 selon Bongiorni *et al*. [2,10]. Le traitement anticoagulant a été aussi incriminé par Lekkerkerker *et al*. [13] en fait ceci serait un facteur indirect de la nécessité de réintervention précoce fortement liée à la majoration du risque infectieux [5,14,15]. Les hématomes de la loge constituent une complication précoce. La revue des essais randomisés a permis de retenir une incidence aux alentours de 2% [16]. Dans notre série, la fréquence de cette complication a atteint 1,53% avec un traitement de fond par AVK comme facteur associé en analyse multivariée. En effet, au cours de la période d'étude, les anticoagulants oraux directs n'étaient pas disponibles en Tunisie et l'arrêt des AVK avec un chevauchement avec l'héparine était une pratique courante [17] adoptée. Ce chevauchement a été incriminé parce qu'il majore le risque hémorragique et qu'il est plus prudent d'implanter sous AVK [17-20] avec un INR voisin de 2. Cette conclusion est précieuse vu le nombre important de patients sous AVK à l'ère où les anticoagulants oraux directs restent chers et non remboursables.

Les complications liées aux sondes étaient les plus fréquentes (73,34%) ce qui est concordant avec les données de la littérature [21]. La cardiopathie hypertensive, le DAI et le nombre de manipulations ont été incriminés, En effet les DAI avec leurs sondes à haut voltage sont plus rigides et plusieurs études ont montré qu'ils sont associés à des complications liées aux sondes plus que les PM [22,23]. Le nombre de manipulations élevé expose aussi aux dysfonctions de sondes et les abrasions de l'isolant [23]. Le déplacement de sonde est ressorti comme la plus fréquente [21] complication majeure, selon les résultats de la première étude prospective et multicentrique (REPLACE) en particulier en cas d'ajout ou de changement de sonde, l'incidence passe de 1 à 7,9%. Le déplacement des sondes est plus fréquent chez les femmes [7,24] surtout obèses [25,26] et en cas de cavités cardiaques très dilatées [27]. Dans notre série, seulement la cardiopathie hypertensive était significativement associée au déplacement de sondes. Les dysfonctions de sondes surviennent le plus souvent au cours de la période d’étude car ils augmentent avec le nombre de manipulations selon notre série. Le taux d'échec de pose de la sonde auriculaire était à 0,21%, celui de la sonde ventriculaire droite 0,43% mais celui de la sonde VG était 7,6%. La fréquence plus élevée avec les sondes VG est expliquée par le faible effectif des patients avec des DEIC triple chambre. Les contraintes anatomiques ont justifié l'échec dans plusieurs situations (hypoplasie du TVBC, remaniements après chirurgie cardiaque, hypoplasie des veines latérales du VG). La perforation cardiaque décrite dans 0,4 à 1% [17,28] était aussi rare mais la plupart des études n'ont pas mentionné cette complication. La rareté des perforations et des complications liées à la voie d'abord témoigne de l'expertise de notre centre. Cependant, la mortalité globale était élevée par rapport aux taux publiés 5,5% à 06 mois selon Kirkfeldt [7], ceci s'explique possiblement par la durée de suivi plus longue et l'âge avancé de notre population.

Par ailleurs, les principaux facteurs dominants de ce travail restent, son caractère rétrospectif et le risque de perte d’informations. Cependant ces patients étaient revus régulièrement, cliniquement et à la télémétrie. Nous avons choisi de recontacter les patients ou leurs médecins pour le complément d'information. Le suivi des patients n'était pas prospectif ce qui pourrait influencer les analyses statistiques. En revanche, cette étude même rétrospective constitue la première analyse de l'expérience de notre centre d'où son intérêt. La notion de dyspnée et de cardiopathie liée à la stimulation du VD n'était pas clairement élucidée car les délais à cette complication n'étaient pas unicistes. L'apprentissage de nouvelles techniques de stimulation plus physiologique est une alternative qui a montré son bénéfice [29,30] par rapport à la stimulation apicale mais elle aurait un spectre de complications différent [31].

## Conclusion

Les résultats de notre centre s'alignent avec ceux de la littérature. Le déplacement de sonde est la complication la plus fréquente et la reprise opératoire expose à d'autres complications. Le taux global des complications reste élevé avec une mortalité significativement plus élevée en cas de complication infectieuse. La détermination des facteurs associés et l'identification de la population à risque comme les diabétiques, les insuffisants cardiaques et les patients sous AVK nous guideraient pour améliorer notre pratique. Eviter le chevauchement héparine AVK et suivre rigoureusement les recommandations de prévention de l'infection sont des mesures primordiales mais la meilleure prévention commence par une indication à bon escient de la stimulation définitive et de son type.

### Etat des connaissances sur le sujet

- Les complications des dispositifs électroniques implantables en cardiologie (DEIC) sont multiples et graves;
- Les données de la littérature concernant les complications de DEIC sont divergentes;
- Les complications infectieuses sont les plus mortelles.

### Contribution de notre étude à la connaissance

- Les taux de complications de la stimulation définitive dans un centre tertiaire au sud tunisien sont semblables aux taux occidentaux, mais la mortalité est plus élevée;
- Les facteurs associés aux complications sont comparables à ceux de l'occident où le diabète, l'insuffisance cardiaque, les AVK et la reprise opératoire sont incriminés;
- La rareté des complications liées à la voie d'accès et des perforations cardiaques témoignent de l'expertise des opérateurs.
